# Rare bacterial isolates causing bloodstream infections in Ethiopian patients with cancer

**DOI:** 10.1186/s13027-017-0150-9

**Published:** 2017-07-11

**Authors:** Balew Arega, Yimtubezinash Wolde-Amanuel, Kelemework Adane, Ezra Belay, Abdulaziz Abubeker, Daniel Asrat

**Affiliations:** 1 College of Health Sciences, Debremarkos University, P.O.Box 269, Debremarkos, Ethiopia; 2 Department of Microbiology, Immunology & Parasitology, School of Medicine, College of Health Sciences, Addis Ababa University, Churchill Avenue, P.O. Box 9086, Addis Ababa, Ethiopia; 3 Department of Microbiology and Immunology, College of Health Sciences, Mekelle University, P.O.Box 1872, Mekelle, Ethiopia; 4 Department Medical Biochemistry, College of Health Sciences, Mekelle University, P.O.Box 1872, Mekelle, Ethiopia; 5 Department of Internal Medicine, School of Medicine, College of Health Sciences, Addis Ababa University, Churchill Avenue, P.O. Box 9086, Addis Ababa, Ethiopia

**Keywords:** Bloodstream infections, Cancer patients, Drug resistance, Ethiopia, Rare bacteria

## Abstract

**Background:**

In recent years, saprophytic bacteria have been emerging as potential human pathogens causing life-threatening infections in patients with malignancies. However, evidence is lacking concerning such bacteria, particularly in sub-Saharan countries. This study was designed to determine the spectrum and drug resistance profile of the rare bacterial pathogens causing bloodstream infections (BSIs) in febrile cancer patients at a referral hospital in Ethiopia.

**Methods:**

Between December 2011 and June 2012, blood samples were collected from 107 patients with cancer in Tikur Anbessa hospital. Culturing was performed using the blood culture bottles and solid media and the microorganisms were identified using the gram staining and APINE identification kits (Biomerieux, France). The disk diffusion method was used for the antimicrobial susceptibility testing.

**Results:**

Overall, 13 (12.2%) rare human pathogens were isolated from 107 adult febrile cancer patients investigated. *Aeromonas hydrophilia* species (a fermentative gram-negative rod) was the predominant isolate, 30.8% (4/13), followed by *Chryseomonas luteola* 15.4% (2/13), *Sphignomonas poucimobilis* 15.4% (2/13), *and Pseudomonas fluorescens* 15.4% (2/13). Of the nine isolates tested for a nine set of antibiotics, 89% were resistant to amoxicillin-clavulanic acid, ampicillin, and trimethoprim-sulphamethoxazole.

**Conclusions:**

This study revealed the emergence of saprophytic bacteria as potential drug-resistant nosocomial pathogens in Ethiopian patients with cancer. As these pathogens are ubiquitous in the environment, infection prevention actions should be strengthened in the hospital and early diagnosis and treatment with appropriate antibiotics are warranted for those already infected.

## Background

Patients with cancer are at a high risk of infection and often the focus of the infection is not apparent. Bloodstream infections (BSIs) have been the leading complications in such patients [1, 2]. In adult patients with malignancies, the crude mortality rates of BSIs range from 18%-42% [3, 4]. A variety of factors including frequent hospitalization, exposure to invasive procedures, use of broad-spectrum antibiotics and chemotherapy make cancer patients more susceptible to BSIs [1, 5]. Chemotherapy renders cancer patients to be neutropenic making them more susceptible to potentially life-threatening BSIs [6, 7].

Bacteria are the primary causative agents of BSIs [8, 9]. The gram-positive pathogens such as *Staphylococcus aureus* (prevalence of *S. aureus* ranging from 18% - 35%) [10, 11] and the gram-negative bacteria such as *E. coil* (13% - 23.1%), *K. pneumoniae* (4.6% - 12%) , and/or *P.aeruginosa* (6% - 12.5%) [10, 12, 13] had been reported as the common pathogens causing BSIs in cancer patients. However, in recent years, saprophytic bacteria have been emerging as potential human pathogens causing life-threatening infections in patients with malignancies. A case report from Delhi State Cancer Institute, for example, indicated that *Aeromonas hydrophila,* a saprophyte in the soil*,* caused a serious soft tissue infection in a woman with breast malignancy [14]. A study from Texas Children’s Hospital isolated the plant pathogen *Pantoea agglomerans* from a blood culture of children [15]*.* Another study from Egypt had also reported the rare bacterial pathogen *Chryseobacterium meningosepticum* from blood cultures of patients with cancer [16].

The spectrum and drug resistance profile of the pathogens causing BSIs in patients with cancer have been reported to show significant fluctuations in different geographical areas and time points [8, 17]. Data regarding the emerging saprophytic potential nosocomial pathogens is limited, particularly in sub-Saharan countries, despite the escalating burden of cancer. This study therefore aimed at determining the spectrum and drug resistance profile of the rare bacterial pathogens causing BSIs in febrile cancer patients at a referral hospital in Ethiopia.

## Methods

### Study setting, design, and populations

This cross-sectional study was conducted at Tikur Anbessa (Black Lion) referral teaching hospital in Addis Ababa, Ethiopia between December 2011 and June 2012. Tikur Anbessa Hospital is the largest hospital in Ethiopia and is a referral site for patients from all parts of the country. To date, this hospital is the only hospital providing cancer chemotherapy and radiotherapy service in Ethiopia. Cancer patients attending the hospital were our study populations. Providing written informed consent, being an age of > 18 years and not being under antibiotic treatment during the study time were the inclusion criteria for this study. All inpatient and outpatient adult febrile cancer patients (*n* = 107) attending the Internal Medicine Department and Oncology-Radiotherapy Center of the hospital during the study period were included in the study conveniently.

### Specimen collection

From each patient included in the study, venous blood was drawn aseptically. Two sets (one-bottle set) of 10 ml for each patient were collected within 24 hours. Then, we poured blood samples into the blood-culture bottles and we incubated them aerobically at 37 °C. We inspected these cultures daily for up to 7 days. Socio-demographic and other relevant data were also collected using structured questionnaire.

### Blood culture and identification

We performed gram staining for broths that showed visible growth (as evidenced by the appearance of turbidity, growing colonies on top of the red cells (‘cotton balls’), hemolysis, and /or gas bubbles). If growth was not detected within 24-hours, we undertook blind sub-culturing to recover pathogenic microorganisms. For bottles that did not show growth until 7 days, we performed terminal sub-culturing. Preliminary identification of the pathogens from the tubes that showed growth was done by gram staining.

Then, we sub-cultured the organisms on Blood agar, Chocolate agar, and MacConkey agar. We incubated these cultures aerobically at 37^0^C and growth was inspected for up to 24-48 hours. We used APINE identification kits (Biomerieux, France) to identify the non-fermentative gram-negative bacilli.

### Antimicrobial susceptibility testing

We used the disk diffusion method for antimicrobial susceptibility testing following standard operating procedures. We prepared suspensions equivalent to a 0.5 McFarland standard by mixing 3-5 bacterial colonies from a pure culture with 5 ml of saline. We then distributed the bacterial suspension evenly over the entire surface of Mueller-Hinton agar using a sterile cotton swab. The inoculated Muller-Hinton plates were left at room temperature for 3-5 minutes to dry and a nine set of antibiotic discs (Oxoid) were dispensed on the surface of each plate. The antibiotics used included amoxicillin-clavulanate (AMC) (30μg), chloramphenicol (C) (30 μg), tetracycline (TTC) (30 μg), trimethoprim-sulphamethoxazole (SXT) (25μg), ceftriaxone (CRO) (30 μg), ampicillin (AMP) (10 μg), nalidixic acid (NA) (30 μg), gentamicin (CN) (10 μg), and ciprofloxacin (CIP) (5 μg). After incubation of the plates at 37^0^C for 24-48 hours, we measured the diameters of the zone of inhibition to the nearest millimeter using a caliper. The isolates were then classified as sensitive, intermediate, and resistant according to the standardized table supplied by the Clinical Laboratory Standard Institute (CLSI). *E. coli* (ATCC-25922) and *P.aeruginosa* (ATCC-27853) were used as standard reference strains for quality control of the culture and antimicrobial susceptibility testing.

### Data analysis

Data were entered using Epi Data entry version 3.1 software and analyzed using SPSS version 16. Descriptive statistics was used to depict the frequencies and proportions.

## Results

### Patient profiles

The study population has been previously described (in press). Briefly, of 107 adult febrile cancer patients enrolled, 52 (48.6%) were males and 55 (51.4%) females with a mean age of 35.5 + 14.64 years. The majority of the study participants, 81 (75.7%), suffered from leukemia and 56 (52.3%) were neutropenic. More than half of the neutropenic patients were with leukemia. Seventy-six (71%) of them had taken cancer therapy in which all received chemotherapy; none had received radiation therapy. About 57% (61/107) had received antibiotics previously of which 64% (39/61) received ceftriaxone, 18% (11/61) ceftriaxone, and vancomycin and the remaining 18% (11/61) received ceftriaxone, vancomycin, and ciprofloxacin. None of the 107 patients investigated had either central intravenous device or indwelling intravenous devices during our investigation, but 76% (81/107) had total parenteral nutrition (TPN) devices.

### Bacterial isolates and their drug susceptibility pattern

Of 107 adult febrile cancer patients investigated for BSIs, 71 bacterial pathogens were isolated of which 13 (18.3%) were rare human pathogens. The characteristics and drug resistance profile of other pathogens have been previously described (in press). Out of the 13 rare bacterial pathogens, the majority 30.8% (4/13) were *Aeromonas hydrophilia* species (a fermentative gram-negative rod). *Chryseomonas luteola, Sphignomonas poucimobilis, and Pseudomonas fluorescens* each accounted two isolates while *Chryseobacterium meningosepticum, Pantoea agglomerans,* and *Serratia ficaria* accounted one isolate for each. The antimicrobial susceptibility results were available for nine of the 13 (69.2%) isolates. Eighty-nine percent of these isolates were resistant to amoxicillin-clavulanic acid, ampicillin, and trimethoprim-sulphamethoxazole (Table 1).

**Table 1:** Susceptibility patterns of the rare bacteria isolated from blood cultures of febrile cancer patients at Tikur Anbessa specialized hospital, Addis Ababa, Ethiopia; December 2011 and June 2012

	Antimicrobial drugs, n (%)									
Organisms		AMC	AMP	C	CRO	SXT	TTC	CIP	NA	CN
*Aeromonas hydrophilia* (*n*=4)	S	-	-	1 (25)	1 (25)	-	-	3 (75)	2 (50)	4 (100)
	I	-	-	1 (25)	-	-	1 (25)	-	-	-
	R	4 (100)	4 (100)	2 (50)	3 (75)	4 (100)	3 (75)	1 (25)	2 (50)	-
*Chryseomonas luteola* (*n*=2)	S	-	-	-	-	-	2 (100)	2 (100)	2 (100)	2 (100)
	I	-	-	-	-	-	-	-	-	
	R	2 (100)	2 (100)	2 (100)	2 (100)	2 (100)	-	-	-	-
*Pseudomonas fluorescens* (*n*=2)	S	-	-	1 (50)	2 (100)		1 (50)	1 (50)	1 (50)	2 (100)
	I	-	-	-	-	-	-	-	-	-
	R	2 (100)	2 (100)	1 (50)	-	2 (100)	1 (50)	1 (50)	1 (50)	-
*Serracia ficaria (n=1)*	S	1 (100)	1 (100)	1 (100)	-	1 (100)	1 (100)	1 (100)	-	-
	I	**-**	**-**	**-**	**-**	**-**	-	-	-	
	R	**-**	**-**	**-**	**-**	**-**	1 (100)	-	-	1 (100)
Total (*n* = 9)	S	1 (11.1)	1 (11.1)	3 (33.3)		1 (11.1)	3 (33.3)	7 (77.8)	5 (62.5)^a^	8 (88.9)
	I	-	-	1 (11.1)		-	1 (11.1	-	-	
	R	8 (88.9)	8 (88.9)	5 (55.6)		8 (88.9)	5 (55.5)	2 (22.2)	3 (37.5)^**a**^	1 (11.1)

^a^= the denominator is 8, *S* Sensitive, *I* Intermediate, *R* Resistant, *AMC* Amoxicillin-clavulanic acid, *AMP* Ampicillin, *C* Chloramphenicol, *CRO* Ceftriaxone, *SXT* Trimethoprim-sulphamethoxazole, *TTC* Teteracycline, *CIP* Ciprofloxacin *GN* Gentamycin, *NA* Nalidixic acid

## Discussion

This study is the first to describe the rare bacterial isolates as causes of BSIs in Ethiopian patients with cancer. The fermentative gram-negative rod, *Aeromonas hydrophilia* species, was the most predominant isolate of the rare bacteria isolated. Previous studies from Egypt had also reported this pathogen as an agent of BSIs in patients with hematological malignancy [18, 19]. *Aeromonas* species are widely distributed in the aquatic environment and patients acquire infection by oral consumption or direct contact with contaminated water or seafood [20]. *Aeromonas* may cause serious fatal infections such as hepatobiliary infection, invasive skin and soft tissue infections, primary bacteremia, burn infections, pleuropulmonary infection, meningitis and endocarditis in the immunocompromised patient [21, 22]. Though *Aeromonas* isolates are susceptible to a broad range of antibiotics, some species may produce beta-lactamase which makes them resistant to ampicillin and first-generation cephalosporins [23]. In this study, all the four isolates were resistant to amoxicillin-clavulanic acid, ampicillin, and trimethoprim-sulphamethoxazole suggesting the emergence of this saprophytic pathogen as a drug resistant potential nosocomial pathogen in Ethiopia.

*Chryseomonas luteola*, a saprophyte found in the soil and water, has only rarely been reported as a human bacterial pathogen [24, 25]. In a case report from a Moroccan University Hospital, this bacterium was indicated causing serious infection in a newborn with a respiratory failure [24]. In the current study, we isolated two *Chryseomonas luteola* pathogens from neutropenic patients with non-Hodgkin lymphoma. In the previous reports, *Chryseomonas luteola* isolates showed variable sensitivity to ampicillin, tetracycline, and co-trimoxazole. In this study, both isolates were resistant to five of the nine antibiotics tested including amoxicillin-clavulanic acid, ampicillin, chloramphenicol, ceftriaxone, and trimethoprim-sulphamethoxazole. This also suggests the emergence of this bacterium as a potential nosocomial pathogen in Ethiopia.

*Chryseobacterium meningosepticum* was isolated from a blood culture of a leukemic patient. This bacterium was already defined as etiology of bloodstream infection among cancer patients by the Infectious Diseases Society of America [26]. A study from Egypt had also reported this bacterium from blood cultures of patients with cancer [16]. *Chryseobacterium meningosepticum* is found in soil and can survive in chlorine-treated municipal water supplies [27] and could colonize patients via contaminated medical devices involving fluids (respirators, intubation tubes, incubators for newborns, ice chests, syringes, intravascular catheters, etc) [28, 29] and cause severe infections in immunocompromised people and in children [16, 28].

Two isolates of *Sphignomonas poucimobilis* were also observed in this study; one from a 40-year-old female patient with acute leukemia and the other from a 24-year-old female patient with chronic lymphocytic leukemia*.* These non-fermenting gram-negative bacilli create a significant problem in clinical settings, being the most widespread cause of nosocomial infections. They are opportunistic pathogens that take advantage of underlying conditions and diseases [30]. According to a review of the case reports by Lin *et al*, about 42 isolates of *S.poucimobilis* were indicated as agents of BSIs in cancer patients (mostly in patients with hematological malignancy) [30]. The existence of this bacterium in our setting could pose a challenge in managing and treating patients appropriately as standard methods to undertake antibiotic susceptibility testing are not on hand for this pathogen [30].

*Pantoea agglomerans,* a very a rare fermentative gram-negative pathogen, was also isolated in our study as an agent of BSIs in patients with acute myeloid leukemia. Previously, this pathogen was isolated from patients with hematological malignancy in Belgium [31]. *Pantoea agglomerans* is a plant pathogen widely distributed in the soil [32]. A review of case reports indicated that *P.agglomerans* was mostly associated with penetrating trauma by vegetative material and catheter-related bacteremia in immunocompromised patients and in children in the hospital [15].

*Serratia ficaria* has been isolated from human clinical samples in rare instances [33]. Although its pathogenicity was always questionable, *S. ficaria* was later found to be able to cause severe infections (septicemia) or deep suppurations such as gallbladder empyema [34]. Thus, *S. ficaria* is an opportunistic pathogen responsible for colonization or serious infections in compromised patients. In this study, we isolated this organism from a 24-year-old female patient with acute lymphocytic leukemia. Another study in France also isolated S*erratia ficaria* from a blood of 83-year-old adenocarcinoma patient [34]. Other case reports [33, 35] also reported this bacterium from a blood culture of patients with underlying disease other than neoplasm. In previous reports, *S. ficaria* was usually susceptible to numerous antibiotics but always resistant to cephalothin (cefazolin was the prophylactic antibiotic in the present case) [34]. In our study, the *S. ficaria* isolate was susceptible to seven of the nine antibiotics tested while it was resistant to teteracycline and nalidixic acid. We didn't test it against cephalothin (cefazolin).

We also recovered two isolates of *Pseudomonas fluorescens* from blood cultures of male febrile neutropenic patients with hematological malignancy. Previously, *P. fluorescens was* reported from a bone marrow transplant patients with hematological malignancies, who became colonized with the organism from a contaminated water dispenser that supplied bottled natural spring water in a hospital [36]. The case holds also true in our setting where the patients didn't have adequate access to clean water supply. A previous study showed that *P. fluorescens* strains isolated from cancer patients were susceptible to gentamicin, neomycin, tetracyclines, polymyxin B, and colistin and resistant to chloramphenicol, ampicillin, and narrow-spectrum cephalosporin [37, 38]. In the current study, both of our isolates were resistant to amoxicillin-clavulanic acid, ampicillin, and trimethoprim-sulphamethoxazole and one of the strains were resistant to teteracycline, ciprofloxacillin and nalidixic acid. This is of some concern because these classes of antibiotics are commonly recommended for the management of neutropenic sepsis in hematological malignancies.

To mention some of the limitations: first we used only aerobic culture techniques; anaerobic microorganisms that might have been contributing for BSIs were not defined. The fact that we took only blood sample might also have limited the range of bacterial pathogens to be isolated.

## Conclusions

This study revealed the emergence of saprophytic bacteria as potential drug-resistant nosocomial pathogens in Ethiopian patients with cancer. The majority of the isolates were resistant to the commonly used chemotherapic antibiotics imposing a challenge to the health care program. As these pathogens are ubiquitous in the environment, infection prevention actions should be strengthened in the hospital and early diagnosis and treatment with appropriate antibiotics are warranted for those already infected.
